# Neuro protective effect of barbiturates leading to successful cerebral recovery after drug induced cardiac arrest and following severe multi organ fail

**DOI:** 10.1186/1757-7241-20-S2-P32

**Published:** 2012-04-16

**Authors:** Mikka Borup, Line Overholt Nielsen

**Affiliations:** 1 Department of anaesthetics, Sygehus Vendsyssel, Hjørring, Denmark; 2 Department of cardiology, Aalborg Sygehus, Denmark

## Background

Barbiturates are known to have a neuro protective effect if given prior to cerebral ischemia. Often in cardiac arrest cases the cerebral ischemia occurs before barbiturates can be administrated.

## Methods

Case report.

## Results

23 year old woman suffered cardiac arrest due to a combined drug overdose with high doses of barbiturates, benzodiazepines and opioids. The patient was found unconscious and after 8 minutes of successful resuscitation transferred to the ICU where she rapidly developed multi organ failure due to the hypoxia and hypo perfusion during the time of cardiac arrest. The organ failure included lever, kidney, lung, pancreas and coagulation, but due to the neuron protective effect of barbiturates, there were no symptoms of cerebral damage. After weeks of stabilizing organ functions the patient was discharged with sustained damage to the kidneys, but no sign of cerebral damage.

## Conclusion

In this case the patients self administration of the barbiturates prior to the cardiac arrest proved to be perfect timed and resulted in an optimal cerebral protection. Brian P. Head et. al (2007) has shown that barbiturates administrated after a hypoxic period still can have a protective effect. Therefore barbiturates can play a crucial role in preventing cerebral damage given both prior to and after ischemic insult.

